# Disseminated Cancer in Pregnancy: Krukenberg Tumour

**DOI:** 10.1155/2014/216969

**Published:** 2014-10-07

**Authors:** Sung-Hee Kim, Siti Rahimah Abd Halim, Naila Siddiqui, Won-Ho Edward Park

**Affiliations:** ^1^School of Medicine, Imperial College London, Exhibition Road, London SW7 2AZ, UK; ^2^Imperial College NHS Trust-NWLH, London, UK; ^3^Imperial College NHS Trust, London, UK

## Abstract

Disseminated cancer presenting in pregnancy is extremely rare and its presentation can easily be obscured by pregnancy and physiological changes that occur with it. This case describes a patient who was diagnosed incidentally following investigations for preeclampsia. Despite initially being thought to be of low risk, the final diagnosis was that of metastasis from a primary cancer of an unknown origin, most likely a gastric adenocarcinoma. Although patients are under thorough observation throughout their pregnancy, this case highlights the potential need for additional investigations or adjustment of current practices. It also draws attention to the lack of sufficient reporting of cancer in pregnancy, which, considering its rarity, greatly influences how patients are managed.

## 1. Introduction

Only one in a thousand pregnancies is complicated by cancers [1]. However, the very state of pregnancy may mask its presentation: there is obstruction by the gravid uterus and the many physiological changes that occur, alongside the commonplace symptoms experienced by the expectant mother including vomiting, bloating, and abdominal distension. Late diagnosis has not only tremendous adverse outcome on the patient's health but also enormous psychological and social implications involving mother and future child. We present a case of disseminated cancer in late pregnancy in a woman with a hitherto normal pregnancy. This serves to illustrate the importance of considering pathology that may be camouflaged by pregnancy as well as highlighting the necessity for improvement in case reporting of malignancy while pregnant.

## 2. Case Presentation

A 27-year-old nulliparous woman was admitted to the obstetrics department with preeclampsia at 29 weeks' gestation. She was following a normal course of pregnancy and complied well with her antenatal schedule, with no abnormalities detected during her visits or ultrasound scans. She was similarly well prior to becoming pregnant with no risk factors that could potentially complicate the pregnancy. The patient had previously been pregnant once, which resulted in a miscarriage, and had a notable past medical history of polycystic ovaries. She did not have a partner and stated that she wished no further involvement of the father in the pregnancy or future care of the baby, wishing to act as a single parent. This pregnancy had come after over a few years difficulty conceiving.

**Figure 1:**
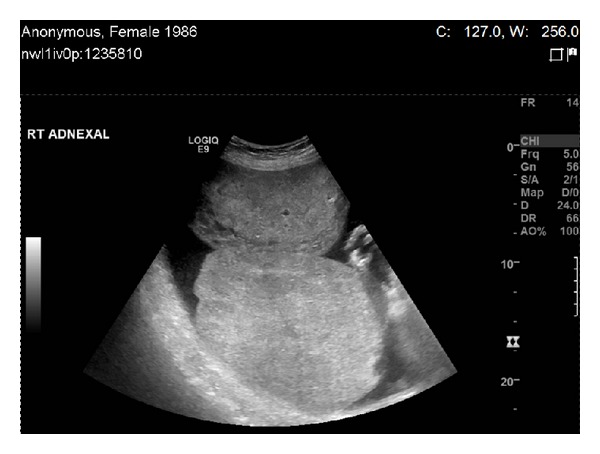
Pelvic ultrasonography revealing a large right-sided adnexal mass of heterogeneous appearance.

**Figure 2:**
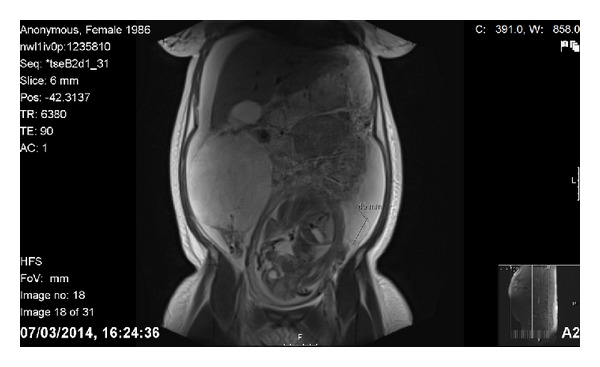
Coronal T2-weighted MR image revealing a gravid uterus with well-defined bilateral ovarian masses and extensive free fluid in the abdomen and pelvis.

**Figure 3:**
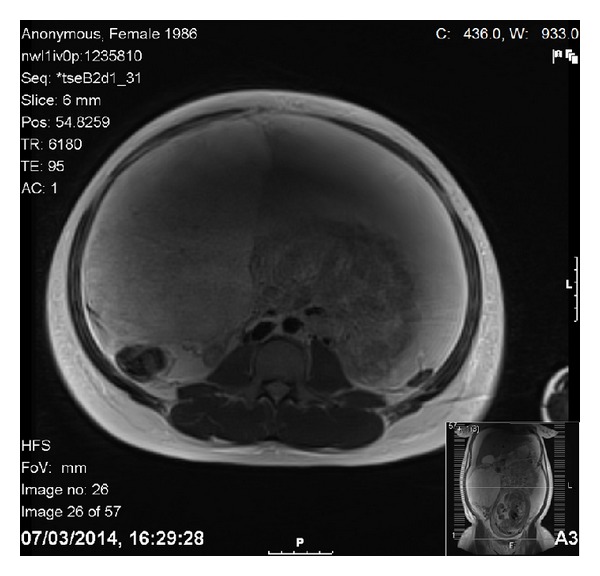
Axial T2-weighted MR image revealing the encapsulated right adnexal tumour.

During the course of her investigations for preeclampsia, she was noted to have ascites on trans-abdominal ultrasound scan and a slight abnormality of her liver function tests (LFT): raised alanine transaminase (ALT). An ultrasound scan and magnetic resonance imaging (MRI) of her pelvis was thus organised which identified a large, well-defined right-sided adnexal mass measuring 15 × 18 × 13.5 cm extending into the upper abdomen, with a vascular pedicle extending from the broad ligament. A smaller mass was also noted in the left adnexa measuring 4.5 cm occurring concurrently with a thickened peritoneum and omentum (Figures 1, 2, and 3). These findings prompted measurements of her tumour markers which demonstrated alpha-fetoprotein 104.3, human chorionic gonadotrophin 14007, lactate dehydrogenase 172, and cancer antigen 125 (CA-125) 1266.

The right-sided mass was connected via its vascular pedicle to the broad ligament/uterine wall but despite a clear spatial relationship, there was little evidence that the tumour was connected to the right ovary. Some images of the right ovary also seemed to be normal. Considering that the patient was largely asymptomatic, this generated a preliminary differential diagnosis of a benign broad ligament tumour with ascites, subserous fibroid with ascites, or a broad ligament fibroma with Meigs' syndrome. We were also mindful that this could be a malignant ovarian tumour.

Initial treatment plans involved a review by the oncology team with repeat radiological assessments as well as awaiting delivery with the intention to perform ovarian cystectomies in order to obtain a diagnosis in the first instance. Paracentesis had also been considered; the benefits of this were not compelling as it would not alter patient management considering that debulking would be a management mainstay of tumours of gynaecological origin. However, shortly following the initial admission, the patient underwent a caesarean section at 31 weeks' gestation due to foetal distress on cardiotocography (CTG) in which the baby was safely delivered. This procedure allowed visual inspection of this pelvis which demonstrated that by this stage, the right mass had progressed into a large 25 cm cystic lesion attached to the broad ligament and posterior wall of the uterus, with an 8 cm irregular ovarian mass on the left. The inspection of the stomach during surgery was unremarkable. Large volume ascites of 3-4 litres was also noted which was drained and sampled for cytology assessment along with omental and peritoneal biopsies.

Following delivery, the patient underwent a right salpingoophorectomy and partial left oophorectomy. Histological analysis of the peritoneum and omentum samples showed infiltration by an adenocarcinoma with areas of signet ring cell differentiation. Immunostaining revealed that the tumour cells were positive for CK7 and CK20. Frozen sections of the ovaries confirmed that this was not high-grade serous ovarian cancer.

The presence of signet cells indicates that the primary malignancy is most likely of gastrointestinal origin and hence that the ovarian masses detected are metastatic, most likely a Krukenberg tumour. This was further supported by the immunochemistry results as tumours that are immunoreactive to CK7 and CK20 suggest a gastropancreatobiliary origin [2]. This conclusion resulted in the decision not to perform a pelvic clearance. Blood results demonstrating deranged clotting and the presence of a large haematoma on caesarean section also made this patient an unsuitable candidate for hysterectomy.

The possibility of a colorectal or lobular breast carcinoma origin had been considered and discussed at both upper GI and gynaecology multidisciplinary team meetings. An endoscopy would ideally have been performed to confirm the working diagnosis, however the patient declined this investigation. As described above, the patient had presented in rapid decline, with life-threatening ascites and pleural effusions. Hence, we were mindful of the window of opportunity to treatment—this would undoubtedly have been narrowed with comprehensive endoscopic investigations. Furthermore, even following debulking, the patient was of Performance Stage 2. Working on the immunohistopathology report which was most in keeping with an upper GI primary, EOX chemotherapy was commenced. This allowed us to begin prompt treatment but also had the added benefit of activity against our differentials which could not, at that stage, be definitively ruled out—oxaliplatin is effective in colorectal cancer and capecitabine is effective in both breast and colorectal cancers.

## 3. Discussion

Malignancy during pregnancy is a rare occurrence. The commonest presentation are of breast cancer (incidence 1 : 3000) [1], followed by cervical, Hodgkin's lymphoma, and ovarian cancers. In our patient, although first suspected to be a cancer of gynaecological origin, the histological finding of signet ring cell differentiation and its bilaterality strongly suggests a Krukenberg tumour: ovarian metastasis from an upper gastrointestinal (GI) origin. Krukenberg tumour, originally described in 1896 [3], accounts for 1-2% of all ovarian tumours [4]. This is a significant finding as only 31 cases of gastric cancers have been reported in pregnant women outside Japan in the last 3 decades, with an additional 103 cases found within the country [5]. More recently, within the past five years only ten cases of Krukenberg tumours during pregnancy have been reported [6–15]. The rarity of this condition is reflected in the paucity of information in the literature regarding guidance for appropriate treatment and management.

Most gastric carcinomas in pregnancy are detected in its advanced stages with histologically poorly differentiated adenocarcinomas—reflected in this patient. Jaspers et al. [5] report that 29% of these malignancies have presented with a Krukenberg tumour. Its late diagnosis is reflected by the poor 3-year survival rate of 8% although this has less of an impact on foetal survival as more than 70% of the pregnancies have resulted in the healthy delivery of the baby. Outside of pregnancy, Krukenberg tumours have a median survival of 14 months [16].

An important point to note is that this patient was considered low risk at booking. She had no known risk factors for GI malignancies given her young age (95% of gastric cancers occur over 50 years of age), no known* H. pylori* infection, and nonsmoker status. Furthermore, she was largely asymptomatic of features suggesting upper GI malignancy, such as dysphagia, and had only started vomiting past 30 weeks' gestation. Despite undergoing multiple ultrasound scans, no pelvic abnormalities had been detected; it was only on presentation of very late symptoms of disseminated disease were adnexal masses and free fluid in the peritoneal cavity identified.

There are two main points of reflection that we would like to draw from this case. Firstly, there is a vital need for earlier detection and diagnosis given the unfavourable outcomes of cases such as this. The diagnosis affects not only the patient but also, inevitably, the quality of life of a newly born baby. Case studies have shown the life changing advantages of earlier detection, allowing the option of curative surgery with much higher survival rates of 50–60% [17, 18]. Furthermore, if the disease is detected before 24 weeks' gestation, it allows the mothers a wider choice of management options including the termination of the pregnancy.

There have been suggestions in the literature for ultrasound visualisation of the ovaries during antenatal scans for early detection of gynaecological malignancies. As scans are already performed in the United Kingdom at least twice during a woman's pregnancy to monitor foetal development [19], this could easily be integrated into current practice. However, the benefits of this change may be questionable. A study conducted by Yazbek et al. [20] of more than 700 women demonstrated that nearly a quarter of all pregnant women had adnexal cysts with the vast majority (85%) resolving spontaneously. Furthermore, no malignancies were found, making ultrasound adnexal visualisation difficult to justify.

Jaspers et al. [5] suggest performing diagnostic gastroscopy in patients complaining of epigastric symptoms in the second trimester, especially if in conjunction with haemoptysis or weight loss. Still, many epigastric symptoms such as nausea and vomiting are disguised as physiological symptoms of pregnancy. This could be overcome by selecting women for further investigation with consideration of their symptoms in combination with existing risk factors, such as, family history of gastric cancer, personal history of gastric ulcer, or heavy smoking.

Existing case reports recommend primary prevention, inducing dietary changes, smoking cessation, and* H. pylori* eradication, as the best management option. Should this preventative measure be insufficient, we would also suggest that following a diagnosis of malignancy in pregnancy, the patient should be promptly transferred to a regional cancer facility that is equipped with a linked obstetrics and neonatal unit. This would greatly improve the care of the patient as, should an emergency caesarean be performed, it would allow simultaneous debulking as well.

Secondly, although further investigations into the nature of the cancer were stalled for the sake of best interests for the patient, there is a concern that there is sparse data available concerning cases such as these. Currently, the National Cancer Registry does not take into account whether the incidence was during a pregnancy. Although the ethics of best interest remain paramount in the medical practice, it is important to consider the value of such information added to the literature and how it would further benefit the management of future patients. In order to overcome such paucity of data, a centralised collection of cases will allow us to have a better insight into managing such conditions and, hence, prevent progression to such dismal outcomes.
